# Plasma glutamine status at intensive care unit admission: an independent risk factor for mortality in critical illness

**DOI:** 10.1186/s13054-021-03640-3

**Published:** 2021-07-07

**Authors:** Marie Smedberg, Johan Helleberg, Åke Norberg, Inga Tjäder, Olav Rooyackers, Jan Wernerman

**Affiliations:** Department of Anaesthesiology and Intensive Care, CLINTEC, Karolinska Institutet and Perioperative Medicine and Intensive Care, Karolinska University Hospital Huddinge Stockholm, B31 Perioperative Medicine and Intensive Care, Karolinska University Hospital Huddinge, 141 86 Stockholm, Sweden

**Keywords:** Plasma glutamine, Critical illness, Hyperglutaminemia, Mortality, Liver failure

## Abstract

**Background:**

A plasma glutamine concentration outside the normal range at Intensive Care Unit (ICU) admission has been reported to be associated with an increased mortality rate. Whereas hypoglutaminemia has been frequently reported, the number of patients with hyperglutaminemia has so far been quite few. Therefore, the association between hyperglutaminemia and mortality outcomes was studied in a prospective, observational study.

**Patients and methods:**

Consecutive admissions to a mixed general ICU were eligible. Exclusion criteria were < 18 years of age, readmissions, no informed consent, or a ‘do not resuscitate’ order at admission. A blood sample was saved within one hour from admission to be analysed by high-pressure liquid chromatography for glutamine concentration. Conventional risk scoring (Simplified Acute Physiology Score and Sequential Organ Failure Assessment) at admission, and mortality outcomes were recorded for all included patients.

**Results:**

Out of 269 included patients, 26 were hyperglutaminemic (≥ 930 µmol/L) at admission. The six-month mortality rate for this subgroup was 46%, compared to 18% for patients with a plasma glutamine concentration < 930 µmol/L (*P* = 0.002). A regression analysis showed that hyperglutaminemia was an independent mortality predictor that added prediction value to conventional admission risk scoring and age.

**Conclusion:**

Hyperglutaminemia in critical illness at ICU admission was an independent mortality predictor, often but not always, associated with an acute liver condition. The mechanism behind a plasma glutamine concentration outside normal range, as well as the prognostic value of repeated measurements of plasma glutamine during ICU stay, remains to be investigated.

**Supplementary Information:**

The online version contains supplementary material available at 10.1186/s13054-021-03640-3.

## Introduction

A lower than normal plasma glutamine at ICU admission is associated with an increased mortality rate. Critically ill patients have an intracellular glutamine deprivation in skeletal muscle [1–3], which has been interpreted as a whole body glutamine deficiency, associated with impairments in important metabolic functions. Enteral nutrition contains glutamine, but conventional intravenous nutrition does not. Therefore, supplementation with glutamine to critically ill patients has been suggested [4]. In particular, addition of a glutamine dipeptide to patients given parenteral nutrition. Several studies on glutamine supplementation of critically ill patients have been published, with variable and sometimes conflicting results [5–7]. Differences in inclusion criteria, doses and route of administration (enteral or parenteral) for the supplementation may partly explain the different results. One proposed mechanism for the negative effects of glutamine supplementation might be the inclusion of patients with high glutamine levels. Very few studies, though, contain information over the actual plasma concentration of glutamine at ICU admission, nor during the intervention. Thus, the original hypothesis that supplementation may be beneficial for subjects with a low plasma glutamine at ICU admission is still not tested [8], and the potential harmfulness of hyperglutaminemia is still obscure.

In parallel with glutamine supplementation for a potential deficit, some investigators suggest using glutamine as immunonutrition. This is derived from the multiple, critical metabolic processes with glutamine involvement, in particular for rapidly dividing cells, with important functions during sepsis and critical illness. Hence, the aim of some studies has not been just to normalize a low plasma glutamine, but rather to obtain pharmacological effects from glutamine supplementation. Moreover, this postulated pharmacological effect has been advocated for the sickest patients, despite the finding that hypoglutaminemia at ICU admission is not related to conventional risk scoring [9–11]. In the largest study investigating glutamine as an immunonutrient, glutamine was given at a very high dose, equalling 70% of the total nitrogen intake [12]. This resulted in a higher mortality in the supplemented group. Unfortunately, this contributed to the current guideline’s advice, not only against using glutamine as immunonutrition, but also against glutamine supplementation for hypoglutaminemia at ICU admission [13].

The mechanism behind a plasma glutamine concentration out of the normal range is still obscure. The low glutamine associated with a high mortality may just be a biomarker without any mechanistic connection. Only a few publications include hyperglutaminemic ICU patients [9–11, 14–16]. A higher than normal plasma glutamine concentration is also reported to be associated with unfavourable outcomes, both at admission and during the course of critical illness [9, 11]. As hyperglutaminemia is frequently observed among patients with liver insufficiency, several cohorts with impaired liver function were recently studied [17]. Acute on chronic liver failure and acute fulminant liver failure are often, but not always, accompanied by hyperglutaminemia, while chronic stable liver failure is not. The prognostic value of hyperglutaminemia in relation to liver failure is therefore not settled.

Although hypoglutaminemia is associated with an increased mortality, the predictive value of admission plasma glutamine concentration outside the normal range (low and high values together) turned out to be an even stronger mortality predictor statistically [9]. The subjects with high glutamine concentration were few in that report, but some of them suffered from liver failure.

In order to better define the possible predictive value of a higher than normal plasma glutamine concentration at ICU admission, we studied consecutive admissions to the ICU with special focus on liver function in a hypothesis generating observational study.

The study had several aims. The primary aim was to correlate the admission plasma glutamine concentration to routine clinical parameters. In particular to enable detection of likely hypoglutaminemia, associated with risk of unfavourable outcomes. This aim was not possible to address properly as the fraction of hypoglutaminemic patients was lower than expected and furthermore in the included cohort hypoglutaminemia was not associated with an unfavourable outcome. The possible reasons for this are put forward in the Discussion section below. Hence, a secondary aim, to further explore the relation between hyperglutaminemia at admission and outcomes as well as comorbidities, diagnoses, and clinical status, is presented here.

## Methods

### Study protocol

In this observational study, consecutive admissions to the general ICU of Karolinska University Hospital Huddinge between 2017-10-01–2018-07-01 were eligible for inclusion.

An extra tube of 4 ml blood was sampled within one hour from admission to the ICU together with routine samples for risk scoring. Informed consent to analyse the sample had to be obtained within one month; otherwise, the sample was destructed. Data on risk scoring, cause of admission, basic characteristics of the patients, and mortality were collected from the patient records. The study is registered at ANZCTR, registration number 379071**.**

The individual result of plasma glutamine analysis was not known by the caregivers and had no impact on medical and nutritional treatment during hospital stay.

### Patients

The general ICU of Karolinska University Hospital Huddinge is a mixed unit with both medical and surgical admissions. Surgical admissions are mainly upper abdominal and liver transplant, while cardiac, neuro, and traumas are handled at the other site of the Karolinska University Hospital. Exclusion criteria were (i) < 18 years of age, (ii) a do not resuscitate order at admission, (iii) readmissions of patients already included into the study and, (iv) absence of informed consent.

Karolinska University Hospital Huddinge is one out of two centres for liver transplantation in Sweden. Most of the patients undergoing liver transplantation are transferred to the ICU post operatively, although the median ICU stay for this cohort is less than 24 h. This group of patients was therefore identified and separated in the post hoc subgrouping (Additional file 1: Table 1B).

### Laboratory analysis

The extra blood sample was collected in an ethylenediaminetetraacetic acid (EDTA) tube, centrifuged within one hour at 2000 xg, and plasma was then stored at − 80 degrees Celsius pending analysis. Glutamine plasma concentrations were analysed as part of a complete aminogram using high-pressure liquid chromatography, as described previously (coefficient of variation 1.6%) [9].

Routine laboratory tests were analysed at the hospital laboratory.

### Statistical analysis

Results are presented as means ± SD or median (interquartile range). Groups are compared with Student’s t-test, Mann–Whitney U-test or Fisher’s exact test as appropriate. Correlations are presented as Pearson’s (r^2^) or Spearman rank correlation (rs). Kaplan–Meier curves and log rank test on survival were performed with IBM SPSS Statistics 24 (Statistical Package for Social Sciences, IBM, NY, USA, 2016). Software Statistica 13.2 (Dell Inc, Tulsa, OK USA) was used to perform univariate logistic regression and multiple logistic regression.

In our previous study, a receiver operating characteristic (ROC) curve for six-months mortality demonstrated a cut-off at plasma glutamine concentration 930 µmol/L or above [9]. Conventional risk scoring, Simplified Acute Physiology Score (SAPS 3) and Sequential Organ Failure Assessment (SOFA), has a high mortality prediction and was therefore included in the regression model. Age was also added as a separate risk factor, although it is already included in SAPS 3, because of its high impact on mortality and was demonstrated as significant also as an isolated predictor in the multiple regression analysis in our previous study [9].

To verify our cut-off for hyperglutaminemia, a ROC curve analysis of patients with a plasma glutamine concentration > 675 µmol/L compared with six-months mortality rate was performed using GraphPad Prism 8 (GraphPad Software Inc., San Diego, CA, USA). The inclusion criterion of values > 675 µmol/L to titrate the lower limit for possible risk was chosen based on the earlier publication [9].

Based on data from our previous study [9], the cut-off for hypoglutaminemia was < 400 µmol/L.

## Results

**Fig. 1:**
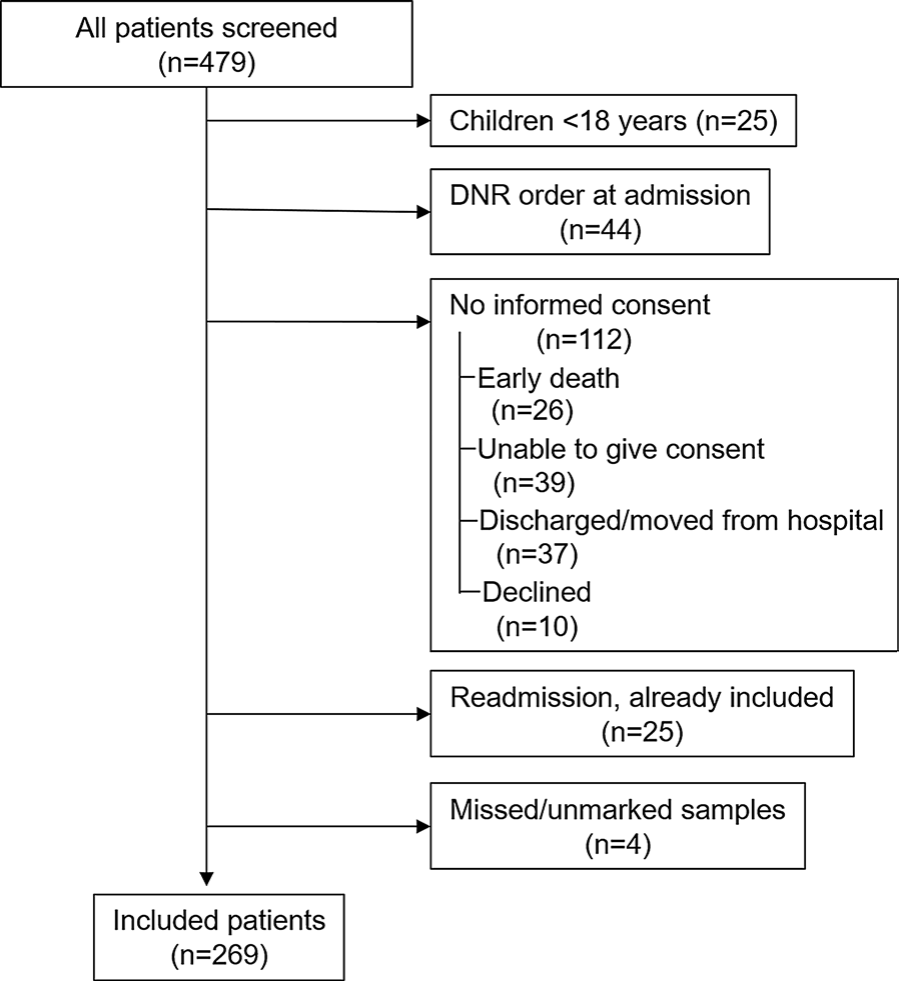
Consort diagram illustrating consecutive patients admitted to the unit during the study period, divided into screened/sampled and excluded patients. DNR = Do Not Resuscitate

During the study period, 504 eligible patients were admitted to the general ICU, 479 patients were sampled, and 269 patients were finally included into the study (Fig. 1). The characteristics of the patients are given in Tables 1 and 2, including the subgroups according to plasma glutamine concentrations, and comorbidities, including liver disease. More extended characteristics are presented in the Additional file 1: Table 1A.

**Table 1:** Main diagnosis at ICU admission related to plasma glutamine concentration at admission

Main diagnosis at ICU admission	p-Gln < 930 µmol/L *n* (%)	p-Gln ≥ 930 µmol/L *n* (%)
Postoperative liver transplant	36 (15)	7 (27)
Liver failure	5 (2)	7 (27)
Post cardiac arrest with ROSC	16 (7)	2 (8)
Kidney failure	11 (5)	0
Respiratory failure	54 (22)	3 (12)
Sepsis/septic chock	38 (16)	2 (8)
Intoxication	17 (7)	0
Diabetic ketoacidosis	5 (2)	0
Cardiac failure/shock	9 (4)	1 (4)
Seizures	9 (4)	0
Major bleeding	11 (5)	2 (8)
Compromised airway	9 (4)	0
Pancreatitis	5 (2)	1 (4)
Circulatory unstable (post-surgery)	5 (2)	0
Esophagus rupture/leak	4 (2)	0
Other	9 (4)	1 (4)

ROSC, return of spontaneous circulation

**Table 2:** Characteristics of subgroups according to plasma glutamine concentration at admission

	All	p-Gln < 400 µmol/L	p-Gln 400–930 µmol/L	p-Gln < 930 µmol/L	p-Gln ≥ 930 µmol/L	*p*
Number of patients	269	40	203	243	26	
ICU mortality	7%	5%	6%	6%	19%	0.097
One-month mortality rate	10%	8%	8%	8%	23%	**0.027**
Six-months mortality rate	21%	20%	18%	18%	46%	**0.002**
SOFA	6 (3–8)	5 (3–8)	6 (3–8)	5 (3–8)	7 (5–11)	**0.006**
SAPS 3	55 ± 16	59 ± 14	55 ± 16	55 ± 16	57 ± 19	0.593
Age (years)	62 (50–71)	65 (52–72)	62 (51–71)	63 (51–71)	52 (37–65)	**0.019**
p-Gln (µmol/L)	585 (469–735)	353 (317–371)	594 (518–700)	557 (456–676)	1165 (998–1473)	**< 0.001**
P-TAA (mmol/L)	2.55 (2.10–3.22)	1.73 (1.51–1.95)	2.59 (2.18–3.04)	2.41 (2.06–2.95)	5.01 (4.14–6.37)	**< 0.001**
Liver disease [*n*, %]	92 (34%)	9 (23%)	61 (30%)	70 (29%)	22 (85%)	**< 0.001**
Kidney failure [*n*, %]	83 (31%)	14 (35%)	58 (29%)	72 (30%)	11 (42%)	0.187

Means ± SD or median and interquartile range

Significant *p* values are indicated in bold

p-Gln, glutamine plasma concentration at admission; SOFA, Sequential Organ Failure Assessment; SAPS, Simplified Acute Physiology Score 3; P-TAA, total amino acid plasma concentration at admission; Liver disease, known liver disease, acute liver failure and/or acute liver damage; Kidney failure, kidney failure according to SAPS scoring and/or creatinine > 180 µmol/L

*p* value indicates the difference between the two groups p-Gln < or ≥ 930 µmol/L.

In the cohort of patients with hyperglutaminemia, the two major diagnoses for ICU admission were postoperative liver transplant and acute liver failure and 85% (22/26) had liver disease and/or signs of acute liver damage at admission.

**Fig. 2:**
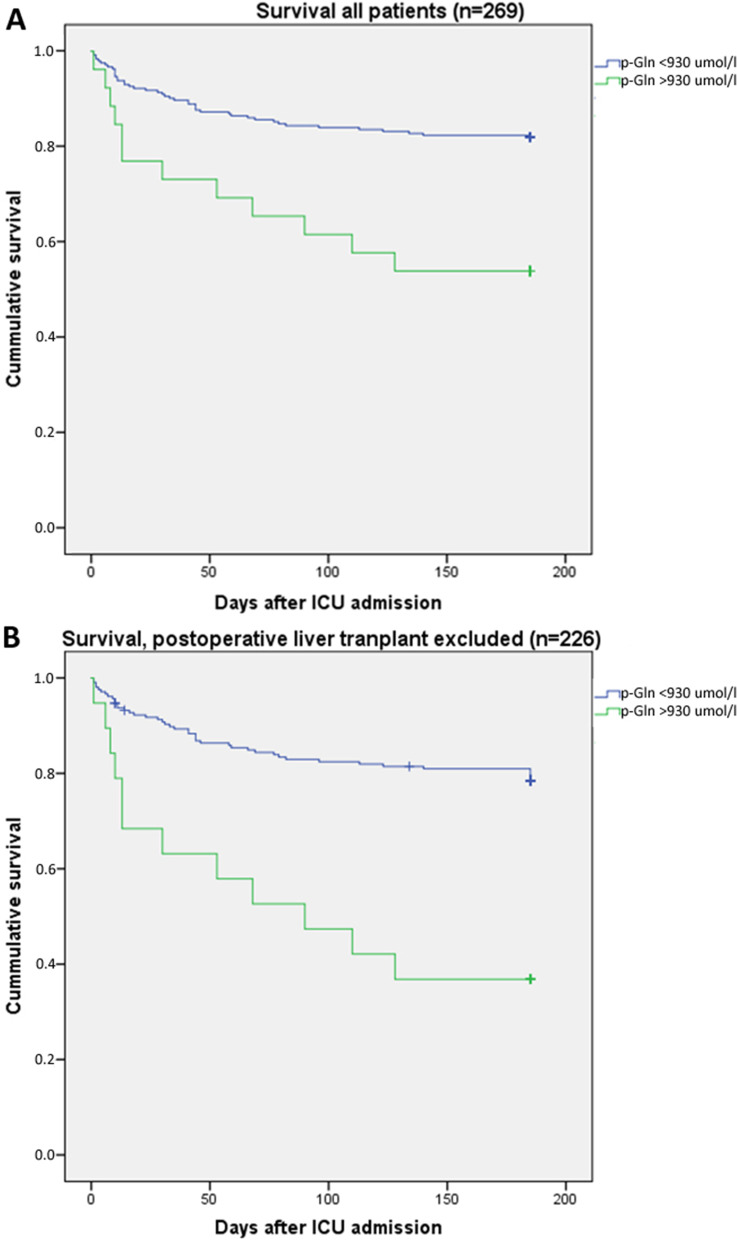
Kaplan–Meier curves illustrating six months survival after ICU admission for patients with plasma glutamine concentration at admission < 930 µmol/L or ≥ 930 µmol/L. Panel **A** shows all patients (*p* = 0.002) and in panel **B** with the postoperative liver transplant patients excluded (*p* < 0.001)

Admission hyperglutaminemia (≥ 930 µmol/L) was associated with a higher mortality rate (*n* = 26, 19% in ICU, 23% at one month, and 46% at six months), as compared to admission plasma glutamine < 930 µmol/L (*n* = 243, 6% in ICU, 8% at one month, and 18% at six months), (*P* = 0.002). This is illustrated in Fig. 2.

Out of the 26 patients with hyperglutaminemia, seven were admissions postoperatively after liver transplantation, and 19 were admitted for other reasons. The mortality rates for these sub-groups with hyperglutaminemia were 0% at six months for post liver transplantation and 21% in the ICU, 32% at one month, and 63% at six months (*P* = 0.01) for the other admission diagnoses. Out of the total cohort of patients included, 43/269 were postoperative after liver transplantation, with a mortality rate of 0% at six months as compared to the mortality rates for the remaining patient group (*n* = 226) of 8% in the ICU, 12% at one month, and 21% at six months.

When a regression model was applied including all subjects (*n* = 269) to predict six months mortality rate, hyperglutaminemia was an independent predictor together with SAPS, admission SOFA and age (Table 3A, B). This finding was stronger when the postoperative liver transplantation subjects were excluded (*n* = 226) (Table 3C, D). Total plasma amino acid concentration was also included in the regression models, but that did not improve them.

**Table 3:** Logistic regression and multiple logistic regression in panels A and B for all patients, and in panels C and D with the postoperative liver transplant patients excluded

Parameter	OR (95% confidence interval)	*p* value
(A) Univariate logistic regression of six-months mortality rate in 269 ICU patients		
p-Gln ≥ 930 µmol/L	1.97 (1.30–2.99)	0.002
Age [per year]	1.038 (1.014–1.063)	< 0.001
SOFA score [per point]	1.18 (1.09–1.27)	< 0.001
SAPS score [per point]	1.042 (1.022–1.063)	< 0.001
All amino acids [per mmol/L]	1.12 (0.96–1.31)	0.059

Parameter	Beta	OR (95% confidence interval)	*p* value
(B) Multivariate logistic regression of six-months mortality rate in 269 ICU patients			
Intercept	− 5.11		
p-Gln ≥ 930 µmol/L	0.779	2.18 (1.26–3.76)	0.006
Age [per year]	0.039	1.040 (1.012–1.069)	0.003
SOFA score [per point]	0.114	1.12 (1.02–1.23)	0.015
SAPS score [per point]	0. 020	1.020 (0.997–1.044)	0.085
All Amino Acids [per mmol/L]	0.006	1.006 (0.876–1.155)	0.932

Parameter	OR (95% confidence interval)	*p* value
(C) Univariate logistic regression of six-months mortality rate in 226 ICU patients		
p-Gln ≥ 930 µmol/L	2.52 (1.54–4.13)	< 0.001
Age [per year]	1.033 (1.010–1.057)	0.002
SOFA score [per point]	1.17 (1.08–1.27)	< 0.001
SAPS score [per point]	1.031 (1.009–1.052)	0.004
All amino acids [per mmol/L]	1.23 (0.98–1.53)	0.015

Parameter	beta	OR (95% confidence interval)	*p* value
(D) Multivariate logistic regression for prediction of six-months mortality rate in 226 ICU patients			
Intercept	− 3.74		
p-Gln ≥ 930 µmol/L	1.14	3.12(1.61–6.06)	< 0.001
Age [per year]	0.046	1.047 (1.018–1.077)	< 0.001
SOFA score [per point]	0.160	1.17 (1.06–1.30)	0.001
SAPS score [per point]	− 0.0056	0.994 (0.968–1.021)	0.679
All amino acids [per mmol/L]	− 0.018	0.98 (0.83–1.16)	0.833

p-Gln, glutamine plasma concentration at admission; SOFA, Sequential Organ Failure Assessment; SAPS, Simplified Acute Physiology Score 3

**Fig. 3:**
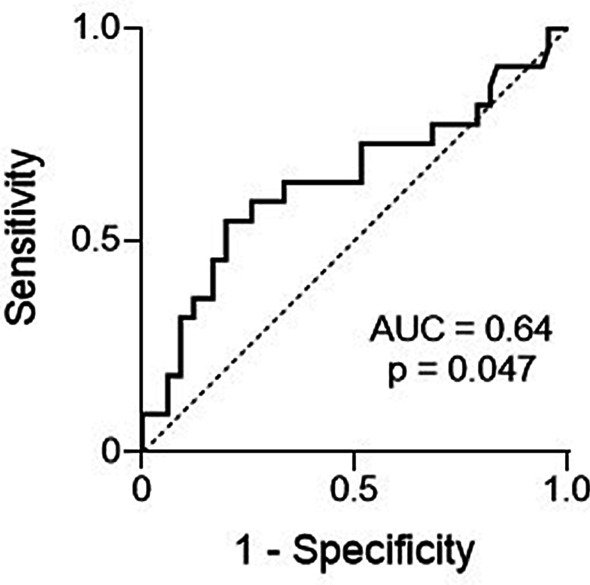
ROC curve of patients with a plasma glutamine concentration > 675 µmol/L compared with six-months mortality (*n* = 88)

A receiver operating characteristic (ROC) curve of patients with a plasma glutamine concentration > 675 µmol/L (*n* = 88) compared with six-months mortality rate is presented in Fig. 3. The area under the ROC curve (AUC) was 0.64 (*p* = 0.047), and the corresponding optimal cut-off value was 932 µmol/L. If the postoperative liver transplanted patients are excluded, ROC AUC was 0.65 (*p* = 0.045) and the corresponding optimal cut-off value was 922 µmol/L (*n* = 62).

## Discussion

The main finding of the study is that hyperglutaminemia at ICU admission was associated with a more than two-fold higher mortality rate at six months compared to patients with normal or low plasma glutamine concentrations at admission. This finding was even stronger when subjects admitted to the ICU postoperatively after liver transplantation were excluded. Furthermore, hyperglutaminemia was a mortality predictor that added prediction value to conventional risk scoring and age.

The inclusion criteria applied in the present study were different from those used in an earlier report, when an association between hypoglutaminemia and a higher mortality rate was demonstrated [9]. The exclusion of subjects with a ‘Do Not Resuscitate’ (DNR) order at admission and, in addition, the absence of informed consent after early deaths in the ICU leading to sample destruction, make the present cohort not comparable to that of the earlier publication, as illustrated in Table 2. The present results, with only 15% of included admissions hypoglutaminemic (< 400 µmol/L), should be contrasted to around one third in earlier reports. Furthermore, the overall six-months mortality rate of the included patients was 21% (58/269), while the estimated mortality rate among the excluded patients would be > 40%, if all patients with DNR orders were assumed dead during the six months follow-up period. So, the selected patient cohort included in the present study was clearly different from other publications [9]. Hence, our present data cannot confirm, but also not contradict, the observations that hypoglutaminemia at ICU admission is associated with an increased mortality rate. This imperfection is unfortunate, but simultaneously it points out the difficulty to compare published patient cohorts, when the inclusion criteria are not meticulously communicated.

The regression analysis showed hyperglutaminemia as a strong mortality predictor also in addition to risk scoring and age. In a selected cohort, as ours, it is common that admission SOFA gives a higher prediction than SAPS 3 due to a stronger reflection of the actual organ failures.

Also, the previous finding of a cut-off for predicting mortality of 930 µmol/L was confirmed, and high plasma glutamine concentration is strengthened as an independent predictor of six-months mortality rate in the ICU setting.

The co-existence of hyperglutaminemia and liver conditions has been shown before [18–22]. It has been connected to the hyperammonemia frequently found in liver conditions, but without any settled mechanism for this observation. Enteral glutamine loading has even been suggested as a diagnostic tool for hepatic encephalopathy [23], however, with limited external acceptance. Up-to-date differentiation of separate cohorts within the group of patients with liver conditions has enabled a more selective view upon the connection between hyperglutaminemia and compromised liver function [17]. In our recent report over plasma glutamine levels in four different cohorts of liver conditions, a general connection between severity of liver disease and plasma glutamine levels was found (AUC for ROC curves 0.75 and 0.82 for Child–Pugh score and Model of End-stage Liver Disease score, respectively) [17]. Hyperglutaminemia was mainly confined to the cohorts with acute fulminant and acute on chronic liver diseases, while chronic liver disease and liver mass reduction by surgery only marginally deviated from normal.

The liver has a crucial role in glutamine metabolism and balance [24]. An impaired liver function can therefore significantly contribute to deranged, high plasma glutamine levels. Accordingly, higher plasma concentrations of bilirubin, alanine aminotransferase, aspartate transaminase, and lactate were associated with high plasma glutamine in our cohort (Additional file 1: table 1A and 1B). Furthermore, it has been suggested that cell damage may result in high plasma glutamine, due to leakage of intracellular glutamine from the destroyed cells into plasma [10], as intracellular glutamine concentrations greatly exceed plasma concentration in many tissues [25–28]. An elevated plasma creatine phosphokinase level as an indicator of cell damage is reported in sepsis patients with high plasma glutamine [10], which supports this hypothesis. Unfortunately, creatine phosphokinase is not a routine sample in our ICU and, therefore, this could not be explored in the present cohort.

It is an interesting observation that the hyperglutaminemic patients undergoing liver transplantation exhibited no mortality during the observation period. Although few in numbers, this observation suggests that a functional liver may be important for normalising the plasma amino acid pattern. Furthermore, in the present cohort of critically ill patients, we find no example where an isolated high glutamine, without connection to a severe pathology, is associated with mortality. The pathophysiology behind the high plasma glutamine levels is likely multifactorial. Glutamine kinetics needs to be further studied in the hyperglutaminemia subgroup to better understand the mechanisms.

There are several limitations in our present study. Most important, the failure to reproduce the connection between hypoglutaminemia and mortality due to the broad exclusion criteria applied, commented upon above. The question is if this may invalidate our main result? We don’t think so, because if the assumption over mortality for the excluded patients is combined with the assumption that none of these individuals were hyperglutaminemic, the hypothetical mortality still comes out statistically different (46% vs. 29%, also with the post liver transplant patients included). Thus, we think that the relatively large number of hyperglutaminemia subjects, make that this report adds value to our understanding of admission glutaminemia, as a biomarker or a reflection of overall glutamine status. The strength of the study is the pragmatic design with virtually no missing data.

In summary, admission hyperglutaminemia (≥ 930 µmol/L) is a prognostic sign for high mortality, which adds predictive value to conventional admission scoring (SAPS 3, SOFA). Hyperglutaminemia is often connected to liver conditions, but is also observed in patients without signs of liver affection. The study is observational in character and measurements of glutamine rates of appearance and disappearance in hyperglutaminemic subjects, as well as repeated sampling during ICU stay, will be needed for better understanding. When plasma glutamine concentration is available in clinical practice and hyperglutaminemia is present, repeated sampling is recommended, and actions should be considered if the level is not decreasing, such as limited protein/amino acid intake.

## Conclusion

Hyperglutaminemia present at ICU admission was an independent mortality predictor for critically ill patients. The presence of hyperglutaminemia was often, but not always, associated with an acute liver condition. In postoperative liver transplanted patients, plasma glutamine concentration was not related to mortality. The mechanism behind an elevated plasma glutamine concentration, as well as the prognostic value of repeated measurements of plasma glutamine during ICU stay, remains to be investigated.

## Supplementary Information

**Additional file 1.** Patient characteristics: Characteristics of high glutamine patients versus normal glutamine patients in all patients (Table 1A) and with newly liver transplanted patients are excluded (Table 1B).

## Data Availability

Extended data on patient characteristics available in pdf Additional file 1.

## Abbreviations

AUC: Area under the curve
DNR: Do not resuscitate
Gln: Glutamine
ICU: Intensive care unit
ROC: Receiver operating characteristic
ROSC: Return of spontaneous circulation
SAPS: Simplified Acute Physiology Score
SOFA: Sequential Organ Failure Assessment
