# Supplementary material for: Plasma glutamine status at intensive care unit admission: an independent risk factor for mortality in critical illness
Source: Crit Care. 2021 Jul 7;25:240. doi: 10.1186/s13054-021-03640-3 (PMC8265095; doi:10.1186/s13054-021-03640-3)
Supplement: Supplementary file 1 — Additional file 1. Patient characteristics: Characteristics of high glutamine patients versus normal glutamine patients in all patients (Table 1A) and with newly liver transplanted patients are excluded (Table 1B). [file 13054_2021_3640_MOESM1_ESM.pdf]

**Table 1A.** Characteristics of high glutamine patients versus normal glutamine patients in 269 ICU patients.

| | p-Gln <930 µmol/L n=243 | p-Gln ≥930 µmol/L n=26 | P |
|---|---|---|---|
| **Age (years)** | 63 (51-71) | 52 (37-65) | **0.019** |
| **BMI (kg/m²)** | 26.7 (23.5-29.9) | 27.9 (25.7-30.6) | 0.155 |
| **SAPS score** | 55 ± 16 | 57 ± 19 | 0.593 |
| **SOFA score (admission)** | 5 (3-8) | 7 (5-11) | **0.006** |
| **Sex [Female: Male]** | 112 : 131 | 13 : 13 | 0.837 |
| **Liver disease [n, %]** | 70 (29%) | 22 (85%) | **<0.001** |
| **Mortality ICU** | 15 (6%) | 4 (19%) | 0.097 |
| **Mortality one month** | 20 (8%) | 6 (23%) | **0.027** |
| **Mortality six months** | 44 (18%) | 12 (46%) | **0.002** |
| **Mortality twelve months** | 52 (21%) | 14 (54%) | **0.001** |
| **Kidney failure [n, %]** | 72 (30%) | 11 (42%) | 0.187 |
| **p-creatinine max day 1 (µmol/L)** | 101 (70-171) | 131 (78-205) | 0.322 |
| **WBC max day 1 (10(9)/L)** | 11.5 (7.6-16.7) | 13.3 (8.9-17.4) | 0.649 |
| **Platelets min day 1 (10(9)/L)** | 179 (112-253) | 178 (90-232) | 0.513 |
| **p-bilirubin max day 1 (µmol/L)** | 12 (6-32) | 65 (16-135) | **<0.001** |
| **Lactate max day 1 (mmol/L)** | 1.7 (1.1-3.5) | 3.2 (2.0-7.3) | **<0.001** |
| **pH min day 1** | 7.35 (7.29-7.39) | 7.32 (7.26-7.38) | 0.189 |
| **p-ALT (µkat/L)** | 1.5 (0.5-9.1) | 13 (3.1-43.8) | **<0.001** |
| **p-AST (µkat/L)** | 2.0 (0.6-11.9) | 15.8 (5.8-40.6) | **<0.001** |
| **p-CRP (mg/L)** | 74 (17-223) | 43 (13-62) | 0.066 |
| **PT (INR)** | 1.4 (1.1-1.6) | 2.0 (1.4-2.9) | **<0.001** |
| **p-urea (mmol/L)** | 7.0 (4.7-11.2) | 7.8 (4.9-12.9) | 0.378 |
| **BE (mmol/L)** | -3.4 (-5.9- -0.6) | -6.0 (-7.8- -3.3) | **0.007** |
| **aB $HCO_3$ (mmol/l)** | 21.6 (19.6-23.9) | 19.4 (17.6-21.8) | **0.006** |

*Means ± SD or median and interquartile range. Lab results from within two hours from admission unless otherwise stated. P-Gln = Glutamine plasma concentration at admission, SAPS = Simplified Acute Physiology Score, SOFA = Sequential Organ Failure Assessment, BMI = Body Mass Index, Liver disease = Known liver disease, acute liver failure and/or acute liver damage, Kidney failure = Kidney failure according to SAPS scoring and/or creatinine >180 µmol/L, max day 1 = maximal value recorded within the first 24 hours, min day 1 = minimal value recorded within the first 24 hours, WBC = white blood cell count, ALT= alanine aminotransferase, AST = Aspartate transaminase, CRP = C-reactive protein, PT = Prothrombin time, BE = Base excess, aB $HCO_3$ = Arterial bloodgas $HCO_3$.*

**Table 1B.** Characteristics of high glutamine patients versus normal glutamine patients in 226 ICU patients. Newly liver transplanted patients are excluded.

| | p-Gln <930 µmol/L n=207 | p-Gln ≥930 µmol/L n=19 | P |
|---|---|---|---|
| **Age (years)** | 63 (51-72) | 52 (37-68) | 0.096 |
| **BMI (kg/m²)** | 26.7 (23.5-30.1) | 28.0 (25.8-33.0) | 0.083 |
| **SAPS score** | 58 ± 15 | 65 ± 14 | 0.055 |
| **SOFA score (admission)** | 6 (3-8) | 7 (4-11) | 0.061 |
| **Sex [Female: Male]** | 91 : 116 | 11 : 8 | 0.336 |
| **Liver disease [n, %]** | 34 (16%) | 15 (79%) | **<0.001** |
| **Mortality ICU** | 15 (7%) | 4 (21%) | 0.061 |
| **Mortality one month** | 20 (10%) | 6 (32%) | **0.012** |
| **Mortality six months** | 44 (21%) | 12 (63%) | **<0.001** |
| **Mortality twelve months** | 51 (25%) | 14 (74%) | **<0.001** |
| **Kidney failure [n, %]** | 72 (35%) | 10 (53%) | 0.139 |
| **p-creatinine max day 1 (µmol/L)** | 109 (72-194) | 150 (78-236) | 0.297 |
| **WBC max day 1 (10(9)/L)** | 12.2 (7.8-17.4) | 13.9 (8.7-17.6) | 0.514 |
| **Platelets min day 1 (10(9)/L)** | 194 (130-268) | 206 (105-255) | 0.993 |
| **p-bilirubin max day 1 (µmol/L)** | 10 (6-21) | 28 (10-76) | **0.006** |
| **Lactate max day 1 (mmol/L)** | 1.7 (1.1-3.2) | 4.8 (2.0-9.0) | **0.001** |
| **pH min day 1** | 7.36 (7.29-7.40) | 7.35 (7.25-7.40) | 0.539 |
| **p-ALT (µkat/L)** | 0.6 (0.4-2.1) | 10.4 (1.1-68.3) | **<0.001** |
| **p-AST (µkat/L)** | 1.0 (0.5-2.4) | 13.1 (3.3-66.6) | **<0.001** |
| **p-CRP (mg/L)** | 84 (16-238) | 37 (13-66) | 0.083 |
| **PT (INR)** | 1.2 (1.1-1.4) | 1.6 (1.1-4.3) | **0.002** |
| **p-urea (mmol/L)** | 7.9 (4.6-13.4) | 11.2 (7.0-13.1) | 0.251 |
| **BE (mmol/L)** | -3.2 (-5.8- -0.3) | -6.0 (-10.9- -2.5) | **0.025** |
| **aB $HCO_3$ (mmol/l)** | 21.9 (19.7-24.2) | 19.4 (15.6-22.4) | **0.027** |

*Means ± SD or median and interquartile range. Lab results from within two hours from admission unless otherwise stated. P-Gln = Glutamine plasma concentration at admission, SAPS = Simplified Acute Physiology Score, SOFA = Sequential Organ Failure Assessment, BMI = Body Mass Index, Liver disease = Known liver disease, acute liver failure and/or acute liver damage, Kidney failure = Kidney failure according to SAPS scoring and/or creatinine >180 µmol/L, max day 1 = maximal value recorded within the first 24 hours, min day 1 = minimal value recorded within the first 24 hours, WBC = white blood cell count, ALT= alanine aminotransferase, AST = Aspartate transaminase, CRP = C-reactive protein, PT = Prothrombin time, BE = Base excess, aB $HCO_3$ = Arterial bloodgas $HCO_3$.*
